# IL-17 in plasma and bronchoalveolar lavage fluid in non-neutropenic patients with invasive pulmonary aspergillosis

**DOI:** 10.3389/fcimb.2024.1402888

**Published:** 2024-08-08

**Authors:** Qian He, Jiaqi Cao, Ming Zhang, Chunlai Feng

**Affiliations:** Department of Respiratory and Critical Care Medicine, Third Affiliated Hospital of Soochow University, Changzhou, China

**Keywords:** invasive pulmonary aspergillosis, IL-17, GM, plasma, bronchoalveolar lavage fluid

## Abstract

**Background:**

The purpose of this study was to investigate the diagnostic value of IL-17 detection in bronchoalveolar lavage fluid (BALF) and plasma samples from nonneutropenic patients with invasive pulmonary aspergillosis.

**Methods:**

We retrospectively collected data on non-neutropenic patients who were suspected to have IPA admitted to the Third Affiliated Hospital of Soochow University between March 2020 to January 2023. IL-17 and GM were measured using enzyme-linked immunosorbent assays.

**Results:**

A total of 281 patients were enrolled in this study, of which 62 had proven or probable IPA and the remaining 219 patients were controls. The plasma and BALF IL-17 levels were significantly higher in the IPA group compared with the control group. The plasma GM, plasma IL17, BALF GM, and BALF IL17 assays had sensitivities of 56.5%, 72.6%, 68.7%, and 81.2%, respectively, and specificities of 87.7%, 69.4%, 91.9%, and 72.6%, respectively. The sensitivity of IL17 in plasma and BALF was higher than that of GM. Plasma GM in combination with IL-17 increases the sensitivity but does not decrease the diagnostic specificity of GM testing. The diagnostic sensitivity and specificity of BALF GM combined with IL-17 for IPA in non-neutropenic patients were greater than 80% and there was a significant increase in sensitivity compared with BALF GM.

**Conclusions:**

Plasma and BALF IL-17 levels were significantly higher in non-neutropenic patients with IPA. The sensitivity of plasma and BLAF IL-17 for diagnosing IPA in non-neutropenic patients was superior to that of GM. Combined detection of lavage fluid GM and IL17 significantly improves the diagnosis of IPA in non-neutropenic patients. The combined detection of GM and IL-17 in plasma also contributes to the diagnosis of IPA in patients who cannot tolerate invasive procedures.

## 1 Introduction

Invasive pulmonary aspergillosis (IPA) is a life-threatening fungal infection predominantly affecting neutropenic patients. These patients generally have severe immune suppression, such as malignant hematological diseases, solid organ or hematopoietic stem cell transplantation, etc (Heylen et al., 2024). In non-neutropenic patients, IPA is relatively uncommon but can occur in individuals with other underlying risk factors, such as chronic obstructive pulmonary disease (COPD) or bronchiectasis (Zhu et al., 2023). The diagnosis of IPA in non-neutropenic patients is challenging due to the lack of specific clinical and radiological manifestations. Traditional diagnostic methods, such as culture and histopathology, have limited sensitivity and specificity, leading to delayed diagnosis and treatment. Therefore, in recent years, there has been a significant increase in research related to the diagnosis of pulmonary aspergillosis in non-neutropenic patients.

GM test is also called *Aspergillus* galactomannan test, which is used to qualitatively detect *Aspergillus* galactomannan *in vitro*, which is released from the tips of *Aspergillus* filaments into blood, bronchoalveolar lavage fluid (BALF) or cerebrospinal fluid during the growth of *Aspergillus* mycelia in tissues (Cai et al., 2023). According to the Infectious Diseases Society of America guidelines (IDSA), GM testing is considered a mycologic criterion for *Aspergillus* infections in neutropenic patients (Patterson et al., 2016). However, GM testing has limited diagnostic value in non-neutropenic IPA patients (Dai et al., 2021). Interleukin-17 (IL-17), which is produced primarily by Th17 lymphocytes, is an early trigger for the induction of an inflammatory response, and it plays a crucial role in host defense against fungal infections by recruiting and activating immune cells, such as neutrophils and macrophages (Puerta-Arias et al., 2020; Launder et al., 2024). Previous studies have found that IL-17 levels in bronchoalveolar lavage fluid from *Aspergillus*-infected mice increased progressively with increasing GM levels (Kimura et al., 2017). In our previous study, plasma IL17 levels have been explored to be significantly higher in patients with IPA and bronchiectasis than in controls (He et al., 2022). However, in patients with fungal lung infections, BALF is a more direct indicator of lung inflammation than blood. In this study, we therefore evaluate the diagnostic value of BALF and plasma IL-17 levels in non-neutropenic patients with IPA.

## 2 Materials and methods

### 2.1 Patients

Non-neutropenic patients who were suspected to have IPA admitted to the Department of Respiratory and Critical Care Medicine of the Third Affiliated Hospital of Soochow University (The First People’s Hospital of Changzhou City) from March 2020 to December 2023 were enrolled in this study. The study was approved by the Institutional Review Board of Changzhou First People’s Hospital. Each patient signed an informed consent form at the beginning of the diagnosis, allowing the use of clinical records for further clinical studies.

The inclusion criteria were as follows:(1) Patients with underlying lung disease such as chronic obstructive pulmonary disease, chronic bronchitis, bronchiectasis, lung cancer, or extra-pulmonary disease including cardiovascular diseases, diabetes mellitus, extra-pulmonary tumors, autoimmune disease. (2) Clinical symptoms of lower respiratory tract infection such as fever, cough, sputum, hemoptysis, or dyspnea, which are not relieved by broad-spectrum antimicrobial drug treatment. (3) Computed tomography of the chest showing solid lung lesions, masses, nodules with or without halo signs, or cavitary lesions.

Cases of “proven” and “probable” invasive pulmonary aspergillosis that meet the diagnostic criteria of the European Organization for Cancer Research and Treatment and the Fungal Disease Research Group (EORTC/MSG) are included in the IPA group (Donnelly et al., 2020). The remaining patients without evidence of invasive *Aspergillus* infection were included in the Non-IPA group, of which 178 patients were finally diagnosed with community-acquired pneumonia(CAP), 35 patients were diagnosed with non-infectious diseases, and 6 patients were finally diagnosed with tuberculosis.

### 2.2 Collection of BALF and plasma samples for the determination of galactomannan and IL-17

Peripheral blood was collected from each patient before the start of treatment. Bronchoscopy was performed by an experienced bronchoscopist. CT was used to localize the diseased bronchial segment or subsegment. The lesions were rinsed twice with 50 ml of saline and BALF was collected in sterile tubes and sent to the laboratory.

The plasma and BALF samples were assayed for *Aspergillus* GM antigen (Platelia *Aspergillus* Kit, Bio-Rad Laboratories, CA, USA) and IL-17 (Human IL-17 ELISA Kit, Abcam) levels by ELISA according to the manufacturer’s instructions.

### 2.3 Statistical analysis

Continuous variables were presented as mean ± standard deviation, while categorical variables were expressed as proportions. Comparisons between groups were performed using the chi-square test or Fisher’s exact test for count data and categorical variables, depending on data distribution. For continuous variables, the Student’s t-test or Mann-Whitney U test was used. Receiver operating characteristic (ROC) curve analysis was conducted to determine the optimal cutoff values for diagnosing IPA using GM and IL17. Statistical analyses were performed using SPSS 23.0 (IBM Corp, Armonk, NY, USA). A significance level of P < 0.05 was considered statistically significant.

## 3 Results

### 3.1 Patient characteristics

**Figure 1:**
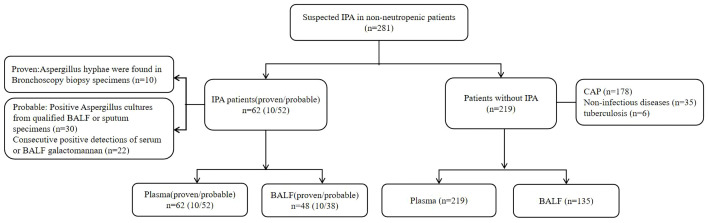
Study fow diagram.

A total of 281 patients were included in this study, of which 62 were diagnosed with IPA (10 proven;52 probable), and the remaining 219 were included in the control group (**Figure 1**).

No significant differences were observed in terms of sex, age, BMI, smoking history, and extra-pulmonary comorbidities between the IPA and control groups. However, In the IPA group, patients with COPD and bronchiectasis were higher than the control group(P<0.05). Similarly, the proportion of patients with steroid treatment in the IPA group was significantly higher than that in the control group (**Table 1**).

**Table 1:** Demographic characteristics of the study population.

Variables	Plasma (n=281)			BALF (n=183)		
	IPA group (n=62)	Control group (n=219)	P value	IPA group (n=48)	Control group (n=135)	P value
Male	37 (59.68%)	133 (60.73%)	0.88	27 (56.25%)	87 (64.44%)	0.31
Age,y	64.5 (54.75,70)	63 (55,71)	0.93	61.5 (53,69.75)	63 (55,69)	0.76
BMI (Body Mass Index)	21.42 (18.95,24.65)	20.90 (19.77,2.28)	0.26	21.42 (18.94,24.95)	20.77 (19.83,22.04)	0.33
Underlying pulmonary diseases						
COPD	20 (32.26%)	37 (16.89%)	0.008	12 (25%)	20 (14.81%)	0.11
Bronchiectasis	18 (29.03%)	35 (15.98%)	0.02	13 (27.08%)	25 (18.52%)	0.21
Previous tuberculosis	10 (16.13%)	49 (22.37%)	0.29	6 (12.5%)	32 (23.70%)	0.1
lung cancer	2 (3.23%)	20 (9.13%)	0.18	1 (2.08%)	14 (10.37%)	0.12
Extra-pulmonary comorbidities						
Cardiovascular diseases	14 (22.58%)	39 (17.81%)	0.4	10 (20.83%)	21 (15.56%)	0.40
Diabetes mellitus	12 (19.35%)	26 (11.87%)	0.13	9 (18.75%)	16 (11.85%)	0.23
Malignant tumor	9 (14.52%)	18 (8.22%)	0.14	6 (12.5%)	8 (5.93%)	0.20
Autoimmune disease	7 (11.29%)	20 (9.13%)	0.61	4 (8.33%)	14 (10.37%)	0.79
History of smoking	28 (45.16%)	75 (34.25%)	0.12	20 (41.67%)	42 (31.11%)	0.18
Steroid treatment	29 (46.77%)	38 (17.35%)	<0.001	18 (37.5%)	22 (16.3%)	0.002

### 3.2 IL-17 and GM levels in plasma and bronchoalveolar lavage fluid

**Figure 2:**
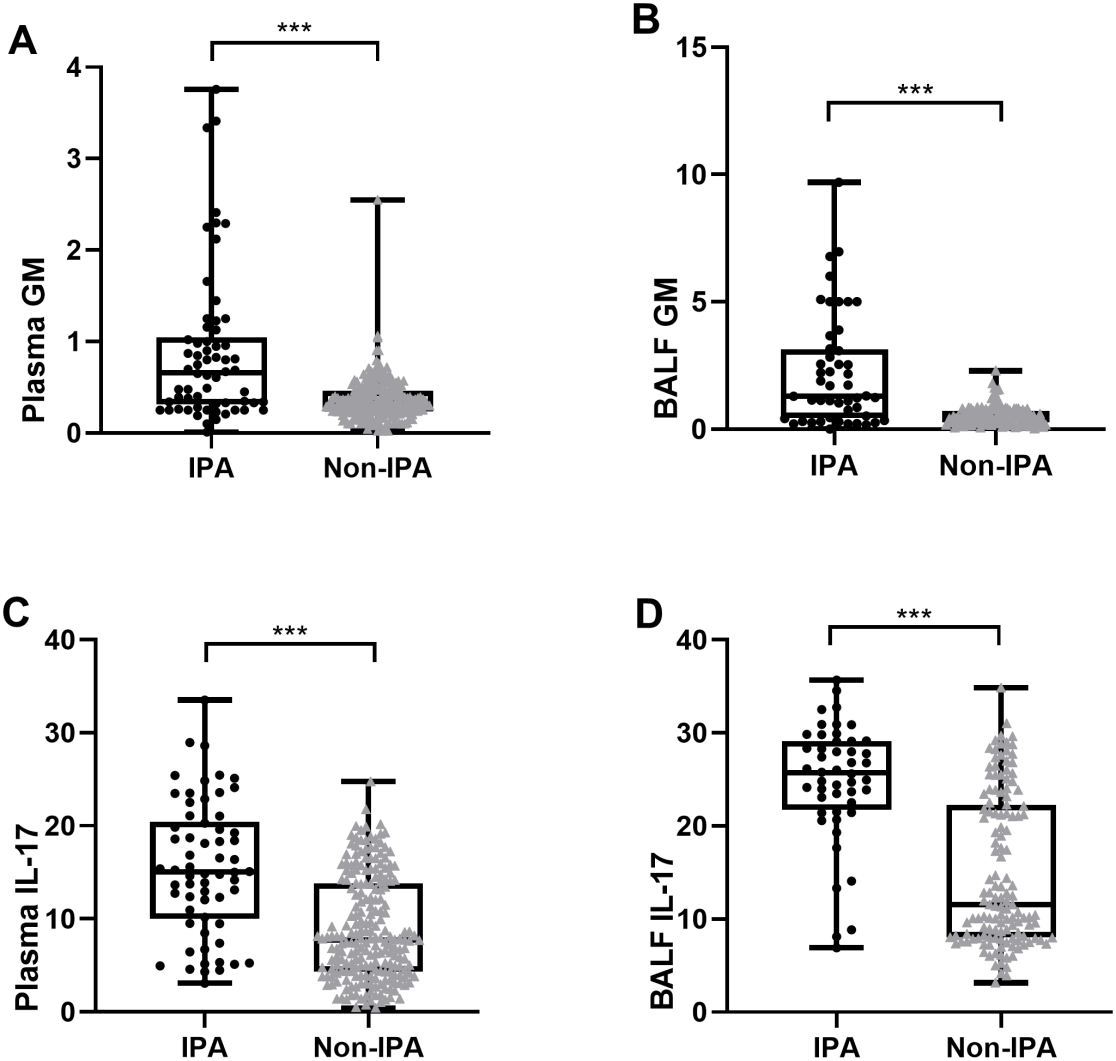
Plasma GM **(A)**, BLAF GM **(B)**, plasma IL-17 **(C)**, BALF IL-17 **(D)** were elevated in IPA group compared with the control group in non-neutropenic patients. ***P<0.001.

The plasma and BALF GM levels in the IPA group were significantly higher than those in the control group (Plasma 0.70[0.33,1.18] vs 0.33[0.25,0.49]; BALF 1.37[0.49,3.12] vs 0.48[0.25, 0.75], P<0.001, **Figure 2**).

The median[IQR] plasma IL-17 levels in the IPA and non-IPA groups were 15.05 [10.00, 20.46]pg./ml and 7.72[4.33,13.79] ng/ml, respectively, the difference was statistically significant (P<0.001); Similarly, the median level of IL-17 in the BALF of the IPA group was significantly higher than that in the control group (25.71[21.76,29.12]pg./mL vs. 11.56[8.03,22.27]pg./mL, P<0.001).

### 3.3 Diagnostic efficiency of IL-17 and GM

According to the ROC curve analysis, the optimal cut-off values for diagnosing IPA were determined as follows. For plasma GM, the optimal cut-off was 0.6, yielding a sensitivity of 56.5% and a specificity of 87.7% (AUC = 0.733). Plasma IL-17 had an optimal cut-off of 12.02 pg./mL, with a sensitivity of 72.6% and a specificity of 69.4% (AUC = 0.756).

In terms of BALF biomarkers, the optimal threshold for BALF GM was identified at 1.01, boasting a sensitivity of 68.7% and a specificity of 91.9% (AUC = 0.786). On the other hand, BALF IL-17 showed a sensitivity of 81.2% and a specificity of 72.6% with a cutoff of 21.32 pg./mL (AUC = 0.811). Notably, despite IL-17 demonstrating lower specificity compared to GM in both plasma and BALF, its sensitivity outperformed GM in accurately diagnosing IPA (**Figure 2**).

### 3.4 Diagnostic accuracy of galactomannan and IL-17 measured in combination

**Figure 3:**
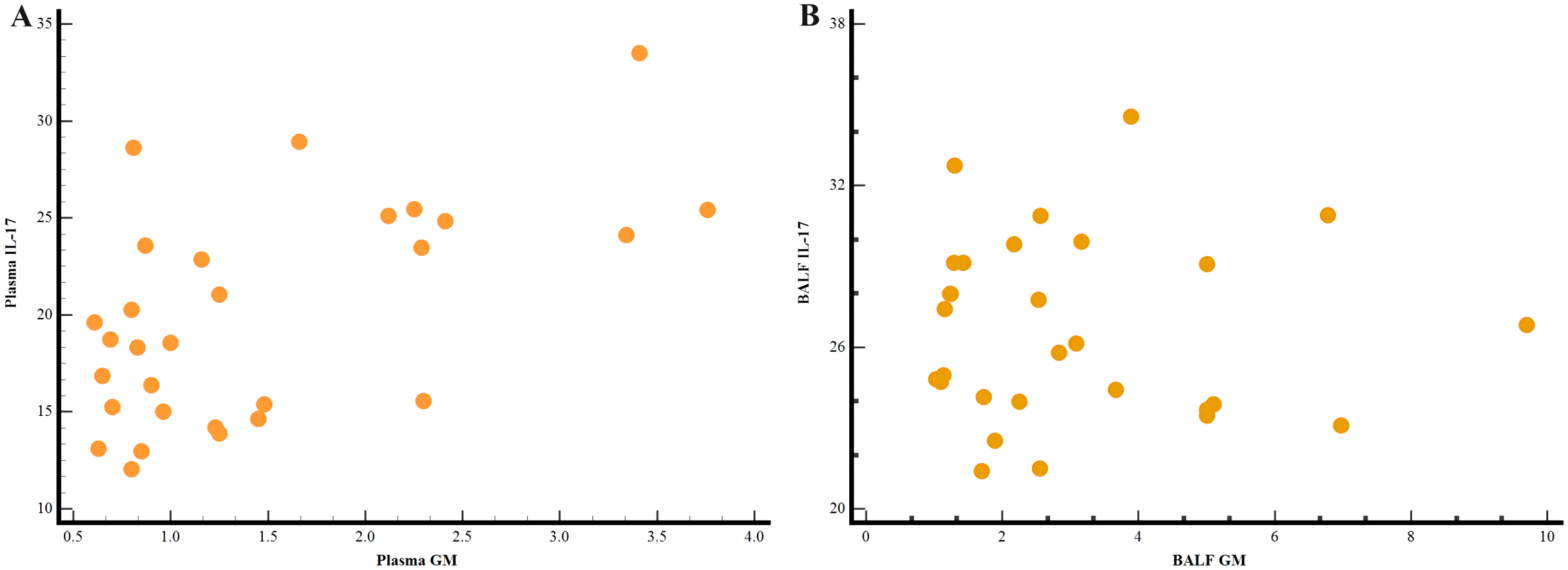
Scatter plot of IPA patients double-positive for IL-17 and GM in plasma **(A)** and BALF samples **(B)**.

We showed the distribution of patients double-positive for GM and IL-17 in plasma and BALF in **Figure 3**. The sensitivity and specificity of double positive plasma GM and IL17 in diagnosing IPA in non-neutropenic patients were 67.7% and 83.1% (AUC=0.816). The area under the curve for the combined diagnosis in plasma samples was superior to that of GM (P=0.011) and IL-17 (P=0.0082).

**Figure 4:**
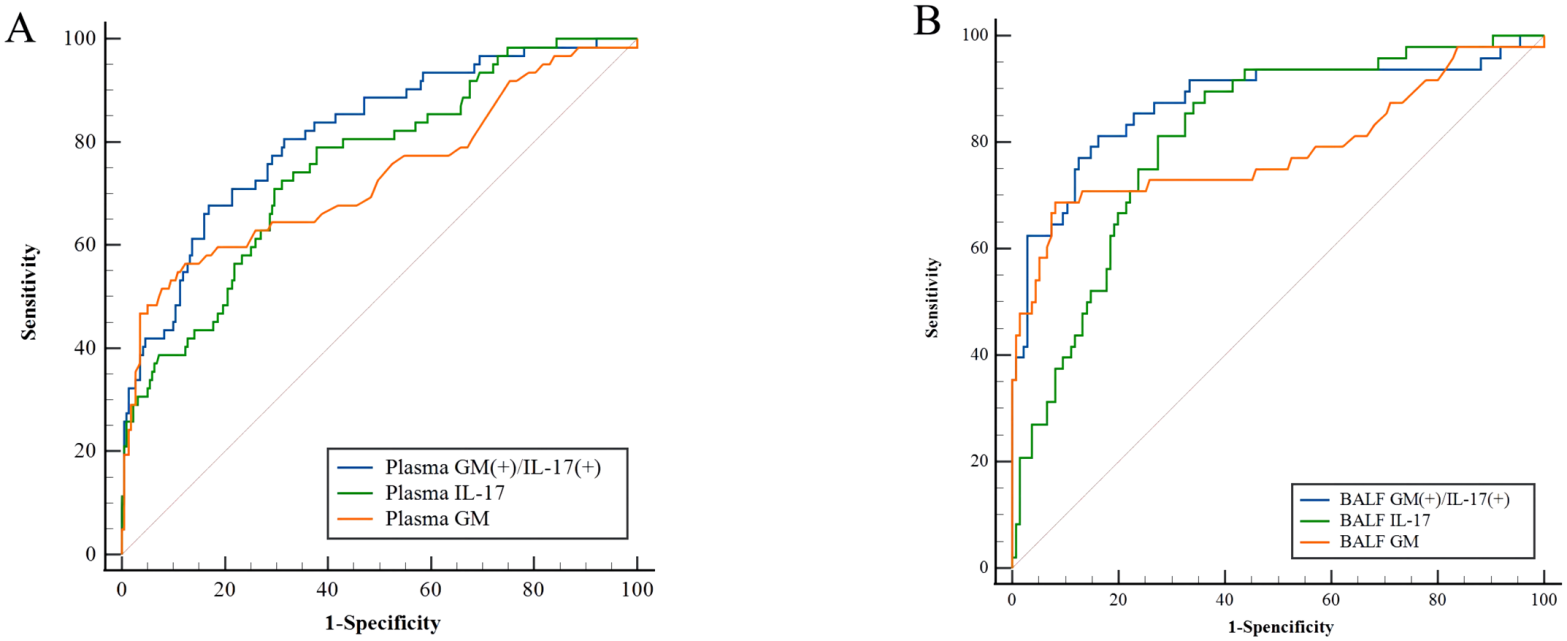
Diagnostic accuracy of GM and IL-17. **(A)** ROC curve for plasma GM/IL-17, The area of plasma GM/IL-17/Double(+) under the ROC curve was 0.733/0.756/0.816, respectively. **(B)** ROC curve for BALF GM/PTX3. The area of BALF GM/IL-17/Double(+) under the ROC curve was 0.786/0.811/0.875, respectively.

In BALF samples, double positivity of the GM and IL17 tests in IPA had a sensitivity of 81.2% and specificity of 83.7% (AUC=0.875). Similarly, the area under the curve of the co-diagnostic in BALF samples was superior to GM (P=0.0047) and IL-17 (P=0.0069). In plasma and BALF samples, the combined diagnosis of GM and IL-17 resulted in a significant increase in sensitivity compared with the GM test (**Figure 4**).

## 4 Discussion

IL-17 is recognized for its crucial role in the innate immune response to invasive aspergillosis infection. Numerous significant studies have emphasized the importance of IL-17 in this context (Khosravi et al., 2018; Malacco et al., 2018). In the present study, IL-17 was more sensitive than GM for the diagnosis of IPA in non-neutropenic patients, both in plasma and BALF, Combined diagnostic testing of GM and IL-17 may improve the diagnostic efficiency of IPA in non-neutropenic patients.

According to the EORTC/MSG recommendations (Donnelly et al., 2020), IPA can be diagnosed by testing plasma and lavage fluid for GM. However, several studies have found that GM testing has limited diagnostic value in in non-neutropenic patients with IPA. In previous studies in non-neutropenic patients, the sensitivity of plasma GM ranged from 11.6% to 90.9% and the specificity from 66.3% to 100% (Zhang et al., 2013; Zhou et al., 2017; He et al., 2021). In this study, the optimal threshold value for analyzing plasma GM based on the ROC curve was 0.6, and the sensitivity and specificity were 56.5% and 87.7%, respectively. In our previous studies, the sensitivity of plasma GM in patients with bronchiectasis was 39.4% and specificity 89.2% (He et al., 2022). The reason for the difference in sensitivity between the two studies is that this study included not only patients with bronchiectasis, but also patients with COPD, previous tuberculosis, etc.

Current research suggests that indicators in BALF are a better surrogate of lung inflammation. In this study, the optimal threshold value of BALF GM for the diagnosis of IPA was 1.01, at which the sensitivity of the test was 68.7% and the specificity was 91.9%. Previous studies have shown that ROC curve analyses of non-neutropenic patients with pulmonary aspergillosis have reported optimal BALF GM cutoff values between 0.7 and 1.25 (He et al., 2012; Zhou et al., 2017; Liu et al., 2021). Our previous study for patients with bronchiectasis combined with IPA reported the same optimal cutoff value of 1.01. In a study in a Chinese population, the optimal cutoff value for diagnosing IPA in non-neutropenic patients with GM in BALF was 0.7 with a sensitivity of 72.97% and a specificity of 89.16% (Zhou et al., 2017). However, in He’s study, the optimal cutoff value for the diagnosis of IPA by BALF GM could be as high as 1.25, which may be due to the fact that the majority of patients included in this study were ICU patients or received mechanical ventilation (He et al., 2012).

IL-17 is primarily produced by a subset of T cells called Th17 cells. Other cell types such as natural killer cells, and neutrophils can also produce IL-17 (Mills, 2023). IL-17 induces the production of chemokines that attract neutrophils to the site of inflammation and enhance the immune response to pathogens (Akhter et al., 2023). It also stimulates the release of pro-inflammatory cytokines such as IL-6 and IL-8, further promoting the inflammatory process (Ritzmann et al., 2022). Current research suggests that IL-17 has emerged as a key factor in host defense against fungal infections (Puerta-Arias et al., 2020). IL-17 promotes the production of antimicrobial peptides such as defensins and cathelicidins, which have direct fungicidal activity against invading pathogens. It can also stimulate the production of chemokines that attract neutrophils and other immune cells to the site of infection, enhancing phagocytosis and clearance of fungal pathogens (Soleimanifar et al., 2023). In an experiment in mice with COPD combined with *Aspergillus* infection, serum IL-17 levels were significantly elevated in *Aspergillus*-infected mice compared with controls, and the lung *Aspergillus* load was almost twice as high in IL-17 knockout mice as compared with controls (Geng et al., 2020). In a study of chronic sinusitis, plasma IL17 levels were found to be significantly higher in patients with combined *Aspergillus* infections compared to controls (Rai et al., 2018). In the study conducted by Hassan et al., elevated serum IL-17 levels were observed in patients with end-stage liver disease who had invasive fungal infections, particularly *Aspergillus* infections (present in 41.67% of cases), compared to those with non-invasive fungal infections. In our previous study, we found that plasma IL17 was significantly higher in IPA patients with bronchiectasis than in controls (Hassan et al., 2014). Our current study is consistent with the above findings. In a study of bronchiectasis combined with *Aspergillus* infection, the sensitivity (78.8%) and specificity (71.9%) at the optimal cut-off value were higher than in this study (He et al., 2022). We believe that differences in the study populations may account for the differences in results. In animal experiments, Kimura et al. observed that levels of IL-17 were markedly increased in BALF from mice infected with *Aspergillus fumigatus*, which correlated with GM levels. Administration of posaconazole resulted in a notable decrease in IL-17 levels in BALF (Kimura et al., 2017). In our study, we found a significant elevation of IL-17 in the BALF compared to the control group. At the optimal cutoff value, the sensitivity of IL-17 in plasma and BALF was significantly higher than that of GM, but the specificity was lower than that of GM.

For patients who cannot undergo bronchoscopy or have difficulty obtaining bronchoalveolar lavage fluid, the sensitivity of diagnosing invasive IPA can be significantly enhanced through combined plasma diagnostics. this study found that the sensitivity (67.7%) of plasma GM combined with IL-17 in diagnosing IPA in patients with non-neutropenic was significantly higher than that of plasma GM, while there was no significant difference in specificity (83.1%). However, the sensitivity of GM combined with IL-17 in diagnosing IPA in patients with bronchiectasis in He’s study (He et al., 2022) (81.8%) was higher than that in this study. The observed differences may be attributed to the following reasons. Firstly, our study included patients with autoimmune conditions who were receiving immunosuppressive therapy, which can affect IL-17 levels. Secondly, there was a significant increase in the number of patients using glucocorticoids in our study compared to previous research(46.77% vs 30.3%), leading to a poorer IL-17 response. Additionally, previous studies exclusively enrolled bronchiectasis patients, who may be more prone to fungal colonization, thus contributing to IL-17 response. In the combined diagnostic assay of BALF GM and IL17, the sensitivity and specificity could reach more than 80%, and there was a significant increase in sensitivity compared with BALF GM. It was also more sensitive than the combined diagnosis of plasma GM and IL17.

Current research suggests that specific antigens of *Aspergillus* can stimulate immune cells in peripheral blood, such as generating antigen-specific T cells (Dellière and Aimanianda, 2023). These specific T cells are capable of producing IL-10, IFN-γ, IL-4, and IL-17A. A study found successful generation of *Aspergillus*-specific T cells in all 8 cases of IPA patients examined, which exhibited cytotoxic subsets capable of lysing *Aspergillus hyphae*. These antigen-specific T cells can be expanded through short-term culture. They primarily target *Aspergillus* cell wall antigens, tend to increase during IPA, and are associated with better clinical outcomes (Potenza et al., 2013). Detecting *Aspergillus*-specific T cells may potentially enhance the diagnosis of IPA. However, in clinical practice, most T cell immune analyses require mononuclear cell isolation, which limits their robustness and practicality, hindering their broader applicability in clinical settings. Therefore, in recent years, researchers have explored a method using dual ɑ-CD28 and ɑ-CD49d co-stimulation to quantify cytokine secretion in response to Aspergillus antigens. This approach strongly enhances the release of T cell signature cytokines induced by Aspergillus, detected through enzyme-linked immunosorbent assay (ELISA). Compared to controls, IPA patients tested using this method exhibited a median concentration of key T helper cell cytokines at least 7 times higher, including IL-17 and Th2 cytokines IL-4 and IL-5, demonstrating the method’s high discriminative capability (Lauruschkat et al., 2021).Therefore, future research will continue to explore the use of antigen-stimulated functional immune assays to enhance the ability to diagnose IPA from peripheral blood samples.

## 5 Conclusions

In conclusion, the sensitivity of IL-17 for the diagnosis of IPA in patients with non-neutropenic was better than that of GM. The combined detection of GM and IL17 in lavage fluid significantly improved the diagnosis of IPA in patients with non-neutrophilic deficiency; in patients who cannot undergo invasive procedures, the combined detection of GM and IL-17 in plasma was also helpful in the diagnosis of IPA.

## Data availability statement

The raw data supporting the conclusions of this article will be made available by the authors, without undue reservation.

## Ethics statement

The study was approved by the Institutional Review Board of Changzhou first people’s Hospital. The studies were conducted in accordance with the local legislation and institutional requirements. The participants provided their written informed consent to participate in this study.

## Author contributions

QH: Conceptualization, Data curation, Formal analysis, Funding acquisition, Investigation, Writing – original draft, Writing – review & editing. JC: Conceptualization, Methodology, Project administration, Writing – original draft. ZM: Supervision, Validation, Visualization, Writing – review & editing. CF: Conceptualization, Investigation, Methodology, Writing – review & editing.
